# Co-expression of *GR79 EPSPS* and *GAT* generates high glyphosate-resistant alfalfa with low glyphosate residues

**DOI:** 10.1007/s42994-023-00119-3

**Published:** 2023-10-19

**Authors:** Yingying Meng, Wenwen Zhang, Zhaoming Wang, Feng Yuan, Sandui Guo, Hao Lin, Lifang Niu

**Affiliations:** 1 Biotechnology Research Institute, Chinese Academy of Agricultural Sciences, Beijing, 100081 China; 2 Inner Mongolia Pratacultural Technology Innovation Center Co., Ltd, Hohhot, 010010 China; 3 National Center of Pratacultural Technology Innovation (Under Preparation), Hohhot, 010010 China

**Keywords:** Alfalfa, *pGR79 EPSPS*, *pGAT*, High-glyphosate-resistant and low-glyphosate-residue, Forage

## Abstract

Weed competition seriously threatens the yield of alfalfa, the most important forage legume worldwide, thus generating herbicide-resistant alfalfa varieties is becoming a necessary cost-effective strategy to assist farmers for weed control. Here, we report the co-expression of plant codon-optimized forms of *GR79 EPSPS* (*pGR79 EPSPS*) and *N-acetyltransferase* (*pGAT*) genes, in alfalfa, via *Agrobacterium*-mediated transformation. We established that the *pGR79 EPSPS*-*pGAT* co-expression alfalfa lines were able to tolerate up to tenfold higher commercial usage of glyphosate and produced approximately ten times lower glyphosate residues than the conventional cultivar. Our findings generate an elite herbicide-resistant germplasm for alfalfa breeding and provide a promising strategy for developing high-glyphosate-resistant and low-glyphosate-residue forages.

**Supplementary Information:**

The online version contains supplementary material available at 10.1007/s42994-023-00119-3.

## Introduction

Alfalfa (*Medicago sativa* L.) is an important perennial forage crop with great economic value owing to its high biomass yield, high nutritional quality as animal feed, nitrogen fixation capacity and wide adaptability to different environments (Bottero et al. 2022). However, alfalfa lacks a competitive advantage compared with weeds, which lead to its yield and quality being seriously threatened. Therefore, exploiting herbicide-resistant alfalfa is becoming important for improving the yield and quality of alfalfa and alleviating this loss (Yi et al. 2018). Genetically modified (GM) herbicide-resistant crops have been grown commercially since 1996, and adopted on a massive scale by North and South America (Duke and Powles 2008). Although not commercialized, transgenic alfalfa, with high resistance to glufosinate-ammonium, was first engineered in 1990 (D’Halluin et al. 1990), GM glyphosate-resistant alfalfa was first transformed in 1998 (McCaslin 2002). In 2005, glyphosate-resistant alfalfa (Roundup Ready®Alfalfa, RRA) was first approved for commercialization in the US, and the complete deregulation of RRA alfalfa was announced by USDA APHIS in 2011 (Heuzé et al. 2016). Recently, multi-herbicide-tolerant alfalfa with tolerance to both sulfonylurea- and imidazolinone-type herbicides were developed by base editing in the acetolactate synthase genes (*ALS1* and *ALS2*) (Bottero et al. 2022). These findings indicated that development of herbicide-resistant alfalfa is an effective strategy for weed control of alfalfa production.

The active herbicide ingredient glyphosate [N-(phosphonomethyl) glycine] is powerful, broad-spectrum, and is widely used for weed control (Beckie 2011; Heap 2014; Liang et al. 2017). Glyphosate inhibits 5-enolpyruvylshikimate-3-phosphate synthase (EPSPS), a necessary chloroplast-localized enzyme of the shikimate pathway, to block the biosynthesis of aromatic amino acids and other secondary plant metabolites (Alibhai and Stallings 2001; Duke 2011; Harrison et al. 1996; Maeda and Dudareva 2012; Pollegioni et al. 2011), leading to eventual plant death. Glyphosate competes with phosphoenolpyruvate (PEP), a substrate of EPSPS, to form a very stable enzyme–herbicide complex that inhibits enzyme catalysis (Schönbrunn et al. 2001). However, unlike plant EPSPS synthase, several microbial EPSPS enzyme variants are not inhibited by glyphosate, such as CP4 EPSPS, G2 EPSPS, G6 EPSPS and G10 EPSPS (Xiao et al. 2019), which have been cloned and used to achieve glyphosate-tolerant transgenic plants (Liang et al. 2017).

It has been shown that GR79 EPSPS, a single amino acid substitution of EPSPS identified from a nitrogen-fixing *Pseudomonas stutzeri* A1501, has high glyphosate tolerance (Liang et al. 2008). Overexpression of plant-codon-optimized *GR79 EPSPS* (*pGR79 EPSPS*) in tobacco, cotton and alfalfa enhanced as tolerance to higher glyphosate than the non-transgenic counterpart (Liang et al. 2017; Yi et al. 2018). Despite the high adoption rate of glyphosate-resistant crops, which could simplify weed management, control weeds effectively and decrease cost–benefit ratio, one of the major disadvantages of glyphosate application is that the residual glyphosate can severely affect plant development of reproductive tissues, resulting in a reduction of crop yield, and is also harmful to the health of humans and animals (Fartyal et al. 2018). Therefore, the detoxification systems for effective removal of accumulated glyphosate residues are used to develop glyphosate-resistant crops with more glyphosate tolerance along with proper plant growth and development.

Many glyphosate-metabolizing bacterial enzymes have been successfully used in crops for generation of glyphosate tolerant transgenic plants (Chen et al. 2023; Fartyal et al. 2018). Among them, glyphosate N-acetyltransferase (GAT) can detoxify the glyphosate by catalyzing acetylation of glyphosate (Liu et al. 2015). Transgenic plants expressing a codon-optimized *GAT* (*pGAT*) gene, identified from soil microorganisms isolated from extremely glyphosate-polluted soil, exhibited significant glyphosate-resistance (Green et al. 2008; Liang et al. 2017). Several strategies have been adopted to achieve a higher tolerance level and minimize the herbicide residuals by co-expressing EPSPS and glyphosate-degradation enzymes (Vennapusa et al. 2022), such as co-expressing *pGR79 EPSPS* and *pGAT* which showed higher glyphosate-tolerance compared with the plants expressing *EPSPS* and *GAT* alone (Dun 2014; Liang et al. 2017). These studies prompted us to examine whether co-expression of *EPSPS* and *GAT* genes could be an effective strategy for developing high-glyphosate-resistant and low-glyphosate-residue alfalfa.

## Materials and methods

### Plant materials and growth conditions

Alfalfa cultivar (*Medicago sativa* L*.* Zhongmu No. 1) was used in the experiments. Clones of wide-type Zhongmu No. 1 and its derived *T*_0_ transgenic alfalfa plants were produced by stem cuttings and grown in an artificial growth chamber at 50% humidity, with a 16 h light (24 °C)/8 h dark (22 °C) photoperiod, or in the experimental field in Langfang (Hebei province, N39°560’, E116°200’).

### Plasmid construction and plant transformation

To generate the constructs used for overexpression, the plant-codon-optimized sequence (CDS) of the *pGR79 EPSPS* (1338 bp) and *pGAT* (441 bp) genes were cloned from the plasmid described previously (Liang et al. 2017). The fragment including *CTP* (*chloroplast-localized signal peptide*)*-pGR79-TNOS* (*NOS terminator*) and *35S:pGAT* was ligated to the pBI121 vector (after digestion with *Bam*HI and *Sac*I) using the In-Fusion cloning system (Clontech, 639648, USA), yielding vector *pBI121-pGR79-pGAT* with the selectable marker gene *neomycin phosphotransferase II* (*NPTII*). The destination construct was introduced into alfalfa variety Zhongmu No. 1 via *Agrobacterium*-mediated transformation as described previously (Jiang et al. 2019). The primers used are listed in Table S1.

### mRNA expression analyses

Total RNA was extracted from the dissected leaves at six-leave stage using TRIzol^™^ Reagent (Invitrogen, 15596018CN, USA) according to the manufacturer’s instructions. Approximately 2 μg of the total RNA was used as a template to synthesize cDNA with EX RT Kit (gDNA remover) (ZOMANBIO, ZR108-2, China). Quantitative RT-PCR was performed as previously described (Meng et al. 2019), using Taq Pro Universal SYBR qPCR Master Mix (Vazyme, Q712-02, China), with three biological and three technical replicates. Gene expression was normalized using the expression of the housekeeping gene *ACC1* (Alexander et al. 2007). All primers used are listed in Table S1.

### Glyphosate tolerance analysis in alfalfa

The glyphosate resistance levels of *T*_0_ generation transgenic alfalfa plants were assessed with glyphosate at a commercial recommended concentration 1200 g a.e./ha (Roundup, PD73-88, USA) at six-leaf stage. For screening the highest glyphosate-resistance level of transgenic alfalfa plants, six-leaf-old clones of wide-type Zhongmu No. 1 and *T*_0_ transgenic alfalfa plants were grown in greenhouse and sprayed with the glyphosate doses of 1200, 4800 and 12,000 g a.e./ha. The phytotoxicity symptoms of transgenic plants were observed in the subsequent 3 weeks. The experiment was conducted with three replicates and each replicate consisted of ten plants. The transgenic plants grown in field in Langfang (Hebei province) were sprayed with 1200 g a.e./ha glyphosate at the six-leaf stage (plant height is about 12 cm). The glyphosate tolerance was evaluated in the following 30 days after glyphosate application (D’Halluin et al. 1990; Liang et al. 2017; Yi et al. 2018).

### Glyphosate residues measured by mass spectrometry

The extraction and measure of glyphosate residues were carried out as previously described with minor modifications (Liang et al. 2017; Zhou et al. 2008). Leaves (500 mg) at the same position of transgenic and wide-type plants were ground into powder in liquid nitrogen, 5 mL water was added and mixed thoroughly. After shaking for 30 min at room temperature, the supernatant was spun in a centrifuge at 5000 rpm for 5 min and transferred to a fresh tube. Then the supernatant was performed with an Oasis HLB solid-phase extraction cartridge (3 mL, 60 mg; Waters). The resulting solution was filtered with a 0.22 μm syringe filter, and analyzed with a liquid chromatography-tandem mass spectrometry system (LCMS-8060, SHIMADZU).

### Agronomic trait analysis

Agronomic traits, including plant height, number of branches and biomass yield were measured on a single-plant basis. The plants grown in greenhouse or in field were analyzed at the six-leaf stage or early flowering stage.

## Results

### Development and characterization of transgenic alfalfa co-expressing *pGR79 EPSPS* and *pGAT*

**Fig. 1:**
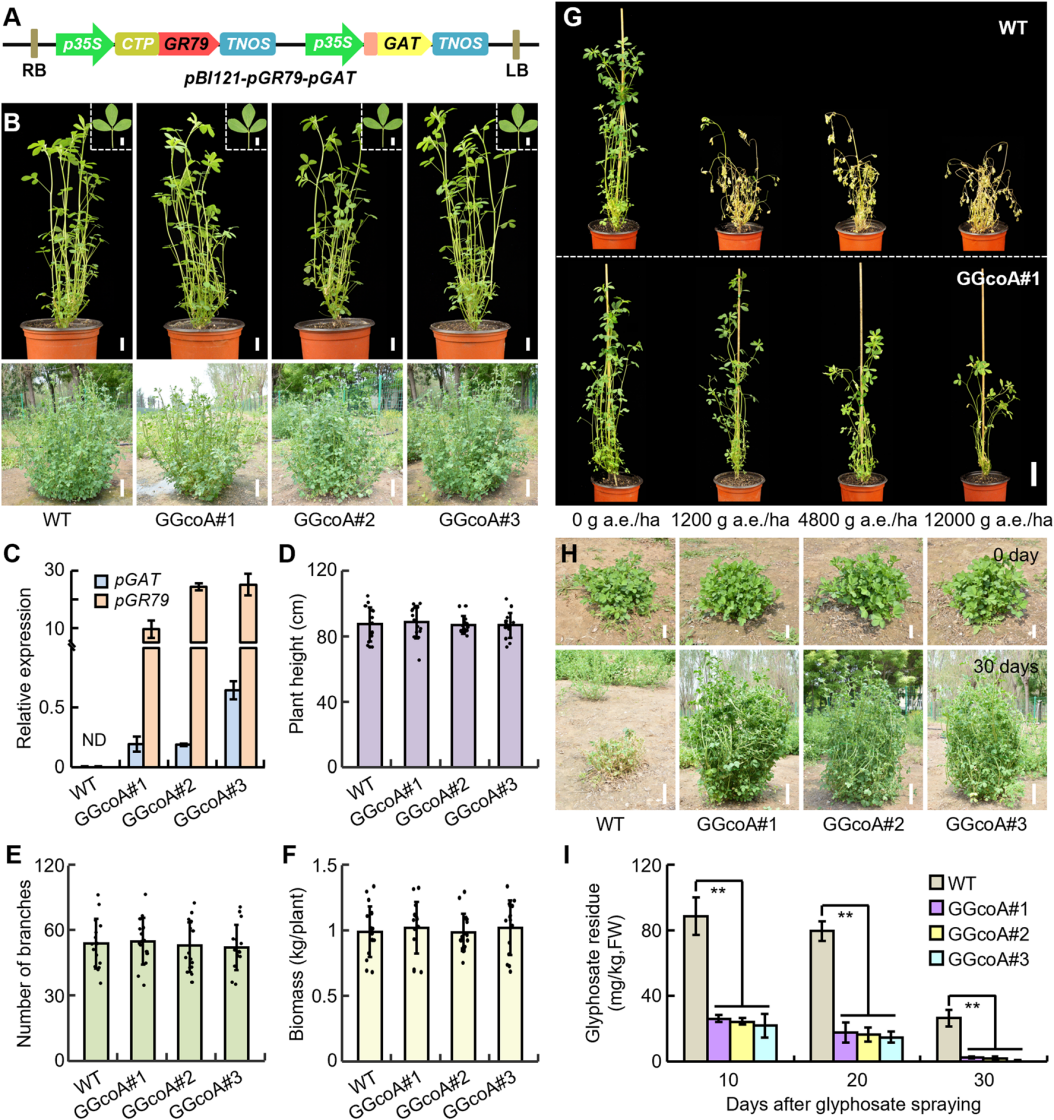
Transgenic alfalfa plants, co-expressing *pGR79 EPSPS-pGAT,* exhibit high herbicide-resistant and low glyphosate residues. **A** Diagram depicting the *pBI121-pGR79-pGAT* construct used in this study. Pink box represents enhancer element. CTP, chloroplast-localized signal peptide. **B** Phenotype of wide type (WT, Zhongmu No. 1) and *pGR79 EPSPS-pGAT* co-expression transgenic alfalfa (GGcoA). The up panel showing six-leaf-stage WT and GGcoA lines grown in greenhouse. Bars, 2 cm; Leaves from the same positions are shown as insets. Bars, 0.5 cm; The bottom panel showing the plants in early flowering stage which were grown on an experimental farm in Langfang (Hebei province), 2023. Bars, 10 cm. **C** Quantitative RT-PCR analysis of the transcript levels of *pGR79 EPSPS* and *pGAT* in leaves of WT and GGcoA lines. The alfalfa *ACC1* gene was used as control to normalize expression levels. Bars represent means ± SD of three biological replicates. ND: not detectable. **D–F** Agronomic traits of WT and GGcoA plants at early flowering stage grown under normal field conditions in Langfang (Hebei province). The plant height (**D**), number of branches (**E**) and biomass (**F**) show no significant difference between the GGcoA plants and WT. Data are means ± SD of ≥ 15 plants. **G** *pGR79 EPSPS-pGAT* co-expression alfalfa plants are resistant to high glyphosate doses. The six-leaf-stage cutting plants of WT and GGcoA#1 grown in greenhouse were sprayed with glyphosate at different doses. The photographs were taken at 7 days after glyphosate application. Bar, 5 cm; **H**, **I** Field evaluations of *pGR79 EPSPS-pGAT* co-expression transgenic alfalfa plants grown on an experimental farm in Langfang (Hebei province), 2023. Glyphosate-resistant phenotype of WT and GGcoAs sprayed with a 1200 g a.e./ha dose of glyphosate (**H**). Photographs were taken at 30 days after glyphosate treatment. Bars, 5 cm for the up panel, 10 cm for the bottom panel. Glyphosate-residue levels in WT and GGcoA leaves were detected after 1200 g a.e./ha doses of glyphosate delivered by spraying (**I**). *FW* fresh weight. Data are means ± SD of three biological replicates; asterisks indicate significant differences from WT (**P < 0.01, Student’s *t* test)

To engineer high-glyphosate-resistant and low-glyphosate-residue alfalfa varieties, we first cloned the plant-codon-optimized *pGR79 EPSPS* and *pGAT* into the pBI121 vector, respectively, obtaining a co-expression construct *pBI121-pGR79-pGAT* (Fig. 1A) (Liang et al. 2017). The N-terminal region of pGR79 EPSPS was fused with a chloroplast transit peptide (CTP), which can precisely and rapidly guide pGR79 EPSPS into chloroplasts where EPSPS functions (Owen 2004). The *pGR79 EPSPS* and *pGAT* co-expression construct was introduced into alfalfa cultivar *Medicago sativa* L*.* (Zhongmu No. 1) by *Agrobacterium*-mediated transformation, and 35 independent transgenic lines were regenerated and verified by PCR with specific primers (Fig. S1). The *T*_0_ transgenic plants grown in the greenhouse were treated with Roundup, at a rate of 1200 g acid equivalents per hectare (a.e./ha) (commercial recommended concentration for alfalfa) (D'Halluin et al. 1990), and three transgenic lines that exhibited significant tolerance to glyphosate (referred to as *pGR79 EPSPS*-*pGAT* co-expression alfalfa, GGcoA) (Fig. 1B) were used for subsequent analyses. Quantitative RT-PCR assays indicated that the three glyphosate-tolerant transgenic lines expressing both *pGR79 EPSPS* and *pGAT*, and the expression of *pGR79 EPSPS* was higher than *pGAT* (Fig. 1C).

To further investigate the glyphosate tolerance of the three GGcoA lines, clonal plants were vegetatively propagated, from each transgenic line, using shoot cuttings. The cutting plants of T_0_ transgenic lines and wild type were grown both in the greenhouse and in an experimental farm in Langfang (Hebei province, N39°560, E116°200), respectively. Several agronomic characters and the glyphosate treatment of the clonal plants were analyzed at the six-leaf stage, or early flowering stage. We determined that there were no significant differences between the three GGcoA (#1, #2, #3) and wild-type alfalfa plants, in terms of plant architecture, leaf size, number of branches, and biomass yield, either grown in the greenhouse or in the field (Figs. 1B, D–F, and S2). These results suggest that *pGR79 EPSPS*-*pGAT* co-expression did not affect the morphological and biomass yield of alfalfa.

### *pGR79 EPSPS*-*pGAT* co-expression transgenic alfalfa showed high-glyphosate-resistant and low-glyphosate-residue

To examine the glyphosate tolerance in the GGcoA transgenic lines, the greenhouse-grown plants at the six-leaf stage were sprayed with glyphosate, at the following doses: 1200 g a.e./ha, 4800 g a.e./ha, 12,000 g a.e./ha. Seven days after glyphosate application, the wide-type plants showed a rapid appearance of chlorosis, necrosis and wilting that led to death, whereas all of the three transgenic lines showed no symptoms at a 1200 g a.e./ha glyphosate dose (Figs. 1G and S3A). Notably, these three GGcoA lines showed marked tolerance to glyphosate, despite the appearance of “yellow flashing” in the young leaves and growth inhibition at 4800 and 12,000 g a.e./ha doses of glyphosate (Figs. 1G and S3A).

To further investigate whether the pGAT enzyme could metabolize glyphosate, we tested the total content of glyphosate in the glyphosate-treated leaves of the GGcoA lines. In comparison to wild type, the co-expression transgenic plants exhibited a rapid and remarkable reduction in glyphosate-residue levels in the leaves 7 days after an application of 1200 g a.e./ha glyphosate. The GGcoA#1, GGcoA#2 and GGcoA#3 plants had 86.6%, 87.3% and 91.2% decreases in glyphosate content, respectively, compared to the wild-type plants. At 14 days post-application, the GGcoA#1, GGcoA#2 and GGcoA#3 plants had 87.1%, 89.4% and 94.5% reductions in glyphosate content relative to the wild-type plants. After 21 days application, the glyphosate concentrations of the GGcoA#1, GGcoA#2 and GGcoA#3 plants were 92.0% (15.7 PPM), 92.5% (14.7 PPM) and 95.5% (8.87 PPM), respectively, lower than that of the wild-type plants (Fig. S3B).

### Field evaluation of *pGR79 EPSPS*-*pGAT* co-expression alfalfa

The glyphosate resistance and residue reduction in the three GGcoA lines were also evaluated in the field. Field evaluations of transgenic plants were investigated for two years in Langfang (Hebei province) in 2022 and 2023. GGcoA and wild-type plants were treated with 1200 g a.e./ha of glyphosate at the six-leaf stage. The leaf and the shoot apical meristems of the wild-type plants were shriveled and exhibited serious herbicide-damage symptoms, shortly after glyphosate applying, and plant growth was completely inhibited. By contrast, all of the three GGcoA lines exhibited no visible damage (Figs. 1H and S4). Similar to low-glyphosate-residue of the greenhouse-growth plants described above, the field-grown co-expression transgenic plants also showed a rapid and significant reduction in glyphosate-residue levels. Especially, at 30 days post-application, the glyphosate concentrations of the GGcoA#1, GGcoA#2 and GGcoA#3 lines were 90.2% (2.6 PPM), 92.0% (2.1 PPM) and 99.2% (0.2 PPM), respectively, lower than that of the wild-type plants (Fig. 1I). Taken together, these results suggest that co-expression of *pGR79 EPSPS* and *pGAT* genes is a feasible and effective strategy for developing high-glyphosate-resistant and low-glyphosate-residue alfalfa.

## Discussion

Glyphosate is a widely used non-selective herbicide with a broad spectrum of weed control around the world. GR79 EPSPS has high catalytic activity and glyphosate tolerance, but a weak affinity for glyphosate (Liang et al. 2008), whereas *GAT* encodes an N-acetyltransferase for acetylation of glyphosate, both of which can offer glyphosate tolerance in GM plants (Castle et al. 2004; Siehl et al. 2005, 2007). Although co-expression of *GR79 EPSPS* and *GAT* genes was reported to confer high tolerance to glyphosate in cotton (Liang et al. 2008), there has been little progress on forages. In this study, we demonstrated that alfalfa lines co-expressing *pGR79 EPSPS-pGAT* were able to tolerate up to tenfold higher commercial usage of glyphosate and produce around 10 times lower glyphosate residues than the conventional cultivar. These results indicate that this combination of genes could be an effective strategy for engineering high glyphosate-resistant alfalfa with low glyphosate residues, which provides a feasible solution for developing elite herbicide-resistant forages.

Nevertheless, it is noteworthy that alfalfa is an autotetraploid, allogamous and self-incompatibility species (Flajoulot et al. 2005), thus further studies are needed to screen homozygous transgenic line with single-copy insertion of *pGR79-pGAT* and evaluate transgene inheritance in order to confer the glyphosate-resistance and low-glyphosate-residue trait in different alfalfa cultivars, by hybridization and molecular-assisted selection. In addition, recent studies have reported the application of genome editing in *EPSPS* for creating glyphosate-resistant crops (Sauer et al. 2016; Li et al. 2016; Jiang et al. 2022). Considering the existence of foreign DNA/genes by transgenic approach, it is worth trying to use genome editing technology to generate transgene-free glyphosate-resistance alfalfa in future studies. Collectively, our findings generate an elite herbicide-resistant germplasm for alfalfa breeding, and provide an attractive and promising strategy for the engineering and breeding of highly resistant low-glyphosate-residue forage varieties.

### Supplementary Information

Below is the link to the electronic supplementary material.

Supplementary file1 (PDF 1704 KB)

## Data Availability

All data generated or analyzed during this study are available from the corresponding author upon reasonable request.
