# Correction: Co-expression of *GR79 EPSPS* and *GAT* generates high glyphosate-resistant alfalfa with low glyphosate residues

**DOI:** 10.1007/s42994-023-00135-3

**Published:** 2024-01-29

**Authors:** Yingying Meng, Wenwen Zhang, Zhaoming Wang, Feng Yuan, Sandui Guo, Hao Lin, Lifang Niu

**Affiliations:** 1 Biotechnology Research Institute, Chinese Academy of Agricultural Sciences, Beijing, 100081 China; 2 Inner Mongolia Pratacultural Technology Innovation Center Co., Ltd, Hohhot, 010010 China; 3 National Center of Pratacultural Technology Innovation (Under Preparation), Hohhot, 010010 China

**Correction: aBIOTECH (2023) 4:352–358** 10.1007/s42994-023-00119-3

In the Acknowledgements section of this article the funding number incorrectly given as SQ2022YFF1000033 and should have been 2022YFF1003204.

The original article has been corrected.

