# Supplementary material for: Co-expression of GR79 EPSPS and GAT generates high glyphosate-resistant alfalfa with low glyphosate residues
Source: aBIOTECH. 2023 Oct 19;4(4):352–8. doi: 10.1007/s42994-023-00119-3 (PMC10721576; doi:10.1007/s42994-023-00119-3)
Supplement: Supplementary file 1 — Supplementary file1 (PDF 1704 KB) [file 42994_2023_119_MOESM1_ESM.pdf]

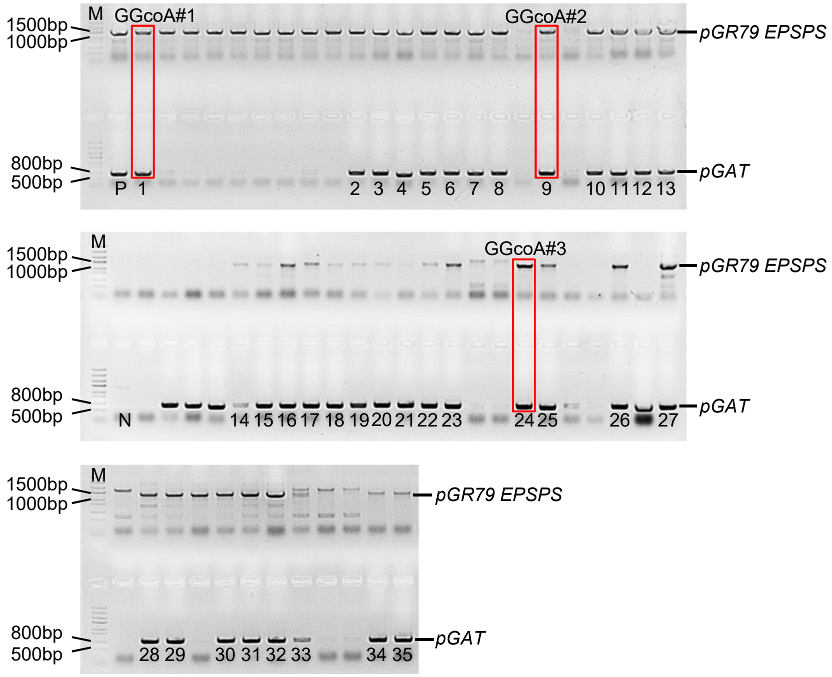

Fig. S1 Identification of the transgenic alfalfa plants containing *pGR79 EPSPS* and *pGAT*.

PCR amplification of *pGR79 EPSPS* and *pGAT* in transgenic alfalfa. The primer pairs of pGR79-jF/pGR79-jR and pGAT-jF/pGAT-jR were used for detecting the *pGR79 EPSPS*, and *pGAT* respectively. 1-35, independent transgenic lines of *pGR79 EPSPS-pGAT* co-expression alfalfa plants; P, positive control, the construct *pBI121-pGR79-pGAT* was used as positive control; N, negative control, the wide type (*Medicago sativa* L. Zhongmu No. 1) was used as negative control; M, DNA marker. Red boxes indicate the three transgenic lines exhibiting significant tolerance to glyphosate.

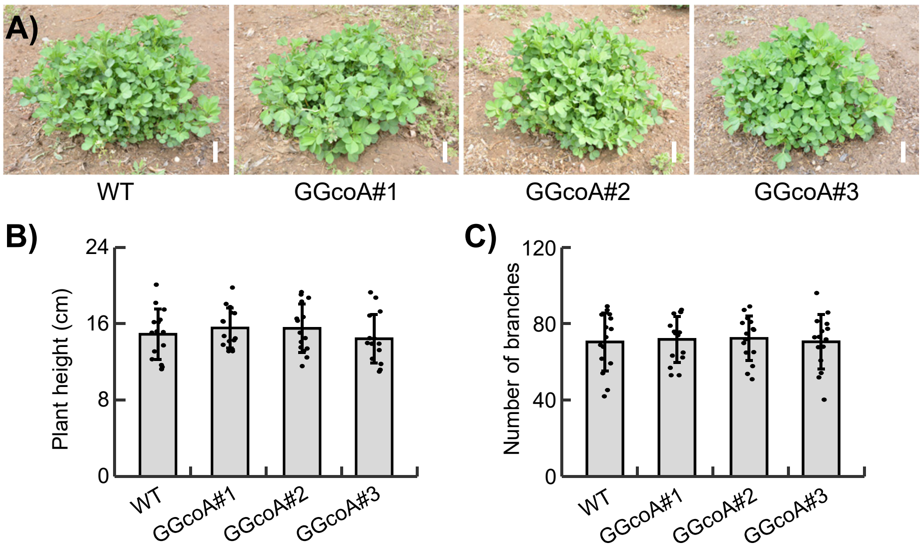

Fig. S2 Phenotype of wild-type (WT) and GGcoA alfalfa plants after overwintering under normal field conditions in Langfang (Hebei province), 2023.

A Seedlings of over-wintered alfalfa WT and GGcoA lines at six-leaf stage. **B-C** The plant height (B) and number of branches (C) of over-wintered WT and GGcoA plants. Data are means ± SD of ≥15 plants.

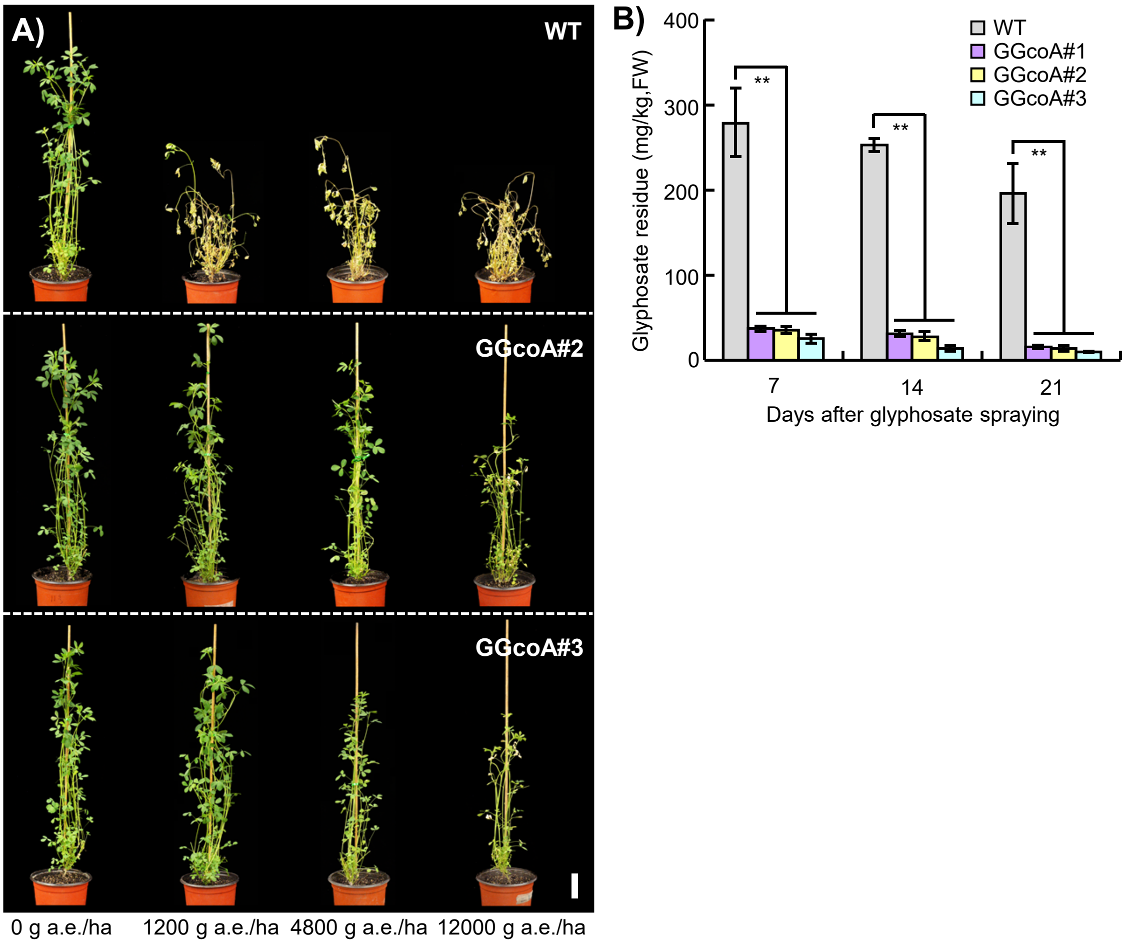

Fig. S3 Transgenic alfalfa plants co-expressing *pGR79 EPSPS* and *pGAT* exhibit high glyphosate resistance and low glyphosate residues.

A Glyphosate-resistant phenotype of *pGR79 EPSPS-pGAT* co-expression alfalfa plants grown in greenhouse. The cutting alfalfa plants at the six-leaf stage were sprayed with glyphosate in 1200 g a.e./ha (commercial concentration), 4800 g a.e./ha, 12000 g a.e./ha doses, respectively. The photographs were taken at 7 days after glyphosate spraying. Bar, 5 cm. The photographs of WT were also showed in Figure 1G. **B** Glyphosate-residue levels in WT and GGcoA leaves were tested after glyphosate spraying. The cutting alfalfa plants at six-leaf stage were sprayed with 1200 g a.e./ha glyphosate, and the leaves for glyphosate-residue measuring were collected at 7, 14 and 21 days after glyphosate spraying, respectively. FW, fresh weight. Data are means ± SD of three biological replicates; asterisks indicate significant differences from WT (** $P < 0.01$, Student's *t*-test).

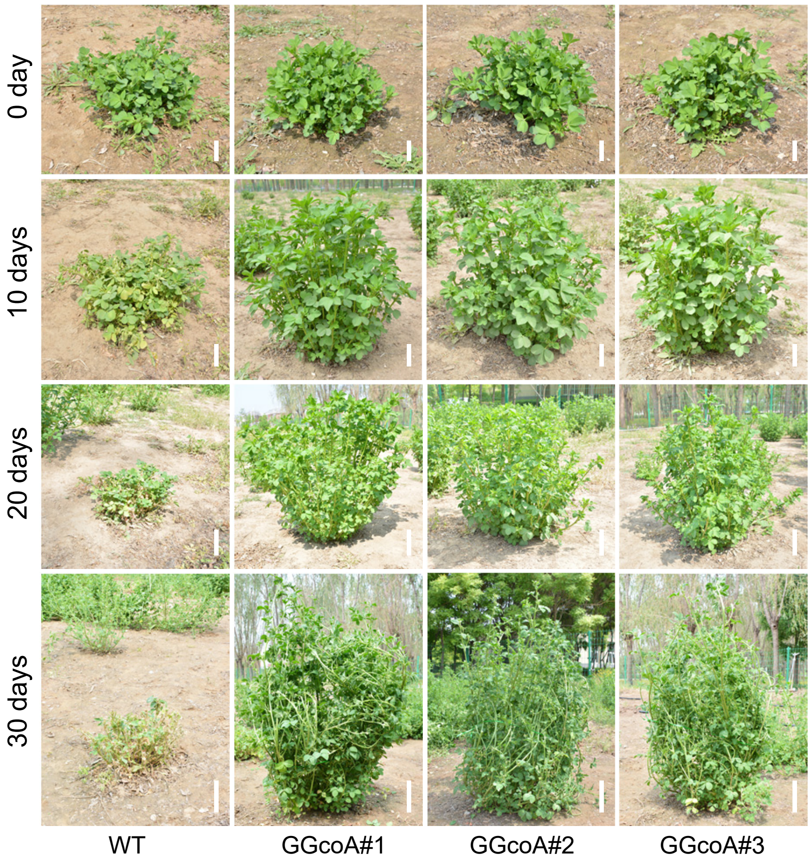

Fig. S4 Field evaluations of *pGR79 EPSPS* and *pGAT* co-expression alfalfa.

Phenotype of WT and GGcoA plants sprayed with 1200 g a.e./ha glyphosate on the experimental farm in Langfang (Hebei province), 2023. The photographs were taken at 0 day, 10 days, 20 days and 30 days after glyphosate spraying. Bars, 5 cm for the first and second panels, 10 cm for the third and fourth panels. The photographs of 0 day and 30 days were also showed in Figure 1H.

Table S1. Primers used in this study

Primer Name	Sequence (5'-3')
**For overexpression experiment**	
CTP-F	GGACTCTAGAGGATCCATGGCAACGCAGTTTGGCAA
pGAT-R	GGGGAAATTCGAGCTCTCAAGCAATTCTCTTATACATCAA
**For genotyping analysis**	
pGAT-jF	ATGATTGATGTGAACCCTATTAAC
pGAT-jR	TCAAGCAATTCTCTTATACATCAAA
pGR79-jF	ATGTCGCATTCCACTTCGCGGTCG
pGR79-jR	CTAATTGTACTCAACGTGGATGCCGAA
**For Quantitative RT-PCR analysis**	
ACC1-qF	GATCAGTGAACTTCGCAAAGTAC
ACC1-qR	CAACGACGTGAACACTACAAC
pGAT-qF	CAGAAACAATATCAGTTGCGTGG
pGAT-qR	ATTACACCAAAGGAGGTCTGCAC
pGR79-qF	AAGGGCGGGTTCGAGGTTGA
pGR79-qR	TGAGCAACGTTAGTGACCCGAATA
